# Remarks on Medical Reform

**Published:** 1812-08

**Authors:** 


*To the Editors of the Medical and Physical Journal.*

GENTLEMEN,

A CORRESPONDENT (H.), in your last Journal, has, I perceive, attempted to revive the old subject of medical reform, and advocate the cause of apothecaries. The purport of his communication lies within a small compass, it appears to be an endeavor to induce practitioners in every part of the kingdom to combine and arrange a plan by which they may extend their emolument without sending in an additional quantity of drugs. This scheme may be very desirable to H. but he offers no argument whatever in its support; and, although the practitioners of Kendal may be ready to unite with him, I very much doubt if his proposal will obtain the general approbation of the profession.

All that he alleges in its favor is, that country practitioners are inadequately paid, unless they throw in a load of medicines, which subjects them to the censure and reproach of their patients. He further insinuates that physicians, though seldom employed, are liberally remunerated; and, if any inference can be drawn from his paper, it is, that apothecaries should receive a fee for their visits or attendance.

Now it appears to me, that, considering the scantiness of education requisite for a country apothecary, the smallness of expence attending it, and the little capital necessary to equip him in his outset, his labor is in general adequately, and, in instances of extraordinary merit, richly, remunerated. Besides a liberal and sufficient profit on his medicines, it is usual to charge visits at a certain distance from his residence, and even in tedious cases, or those which occupy much of his time, attendance left blank in the bill commonly obtains for him a very liberal present.

The certain consequence of this high premium for medical practitioners of moderate attainments is, that the number of competitors greatly exceeds the demand, and this circumstance alone is sufficient to counteract the proposition of H. because, more practitioners being in the market than can be employed, some of them must offer their services at a lower rate; and, where there is not much variation in the scale of ability, he who will perform his business at the lowest rate will acquire the greatest share of practice; consequently the scheme of H. if enforced, would ruin those practitioners who adopted it, and throw business into the hands of those who previously had none. Or, supposing for a moment that the plan was acted upon by the whole profession, it would induce people to pause before they sent for a practitioner at a certain and enormous expence; they would either, as indeed is becoming very common, get an occasional visit from a physician, or quack themselves out of some of the modern domestic medicine books.

Now we well know that an apothecary is frequently long in attendance, and derives great part of his income from cases in themselves trivial, and which, if the fee was augmented, the patients might find, would soon recover if not interfered with. On these grounds I firmly believe that a very great majority of country practitioners will reject any innovation in the usual mode of remunerating their services, whether it proceed from Westmoreland or from Lincolnshire. These things are much better left to individual management. The practitioner, who in the country visits his patients in a chariot, knows how to obtain payment in proportion to his increased increased expenditure, without the interference of a society or an association.

H. talks of the remuneration of physicians: I know not how it is in London, but in the country I am convinced they are worse paid than any other description of practitioners; seldom called in but at the death; and, with a heavy deduction of chaise-hire, very few indeed obtain that income which their education and rank in life demand.

I am, Gentlemen,

*Yorkshire,* Your very obedient Servant,

*June* 15, 1812. *A contented Country Practitioner.*

